# Supramolecular assembly of phenanthrene–DNA conjugates into light-harvesting nanospheres†‡

**DOI:** 10.1039/d4nj02411g

**Published:** 2024-08-30

**Authors:** Jan Thiede, Thomas Schneeberger, Ioan Iacovache, Simon M. Langenegger, Benoît Zuber, Robert Häner

**Affiliations:** a Department of Chemistry, Biochemistry, and Pharmaceutical Sciences, University of Bern Freiestrasse 3 CH-3012 Bern Switzerland robert.haener@unibe.ch https://www.haener.dcbp.unibe.ch; b Institute of Anatomy, University of Bern Baltzerstrasse 2 CH-3012 Bern Switzerland

## Abstract

The self-assembly of highly functionalized phenanthrene–DNA conjugates into supramolecular nanostructures is presented. DNA oligomers modified with phenanthrene residues at the 3′-end and internal positions self-assemble into spherical nanostructures. The nanospheres exhibit light-harvesting properties. Upon irradiation of phenanthrene, the excitation energy is transferred along phenanthrene units, resulting in phenanthrene–pyrene exciplex formation.

## Abstract 2

The self-assembly of DNA–chromophore conjugates into nanospheres is demonstrated. Phenanthrenes are arranged in light-harvesting chromophore arrays collecting excitation energy *via* FRET.

## Graphical abstract

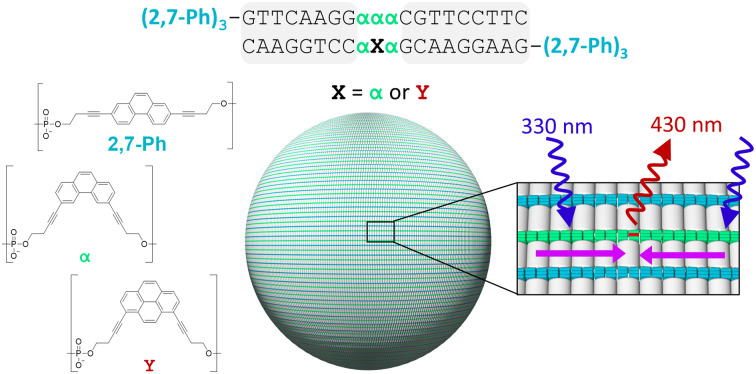

## Introduction

DNA nanotechnology is a rapidly advancing domain of study wherein DNA serves as the building material for precise nanostructure assembly.^1–7^ The rigid three-dimensional scaffold of the DNA double helix enables the creation of spatially and functionally predefined architectures.^8–11^ The most widely used approach to form DNA-constructed nanostructures is DNA origami.^12–14^ An alternative approach consists in the chemical modification of DNA with artificial building blocks.^15–19^ In contrast to the DNA origami approach, these DNA conjugates self-assemble *via* supramolecular interactions of the modifications, which greatly extends the range of DNA-enabled nano-assemblies. A straightforward approach is the introduction of hydrophobic (sticky) ends.^20–26^ Hydrophobic interactions among the sticky ends lead to the self-assembly of the modified DNA conjugates into nanostructures. DNA conjugates modified with hydrophobic chromophores are of significant interest, as they can simultaneously enable self-assembly and function as light-harvesting antennas by collecting and transferring excitation energy.^27–29^ In addition, the structural features of DNA also allow a precise arrangement of chromophores, which proved pivotal for the formation of artificial light-harvesting complexes (LHCs).^30–37^

Previously, we reported on the supramolecular self-assembly of DNA conjugates modified at the 3′-end, forming nanostructures using phenanthrene, tetraphenylethylene, or pyrene sticky ends.^27–29^ The hydrophobic interactions between the aromatic appendages play a crucial role in the self-assembly process. In the presence of spermine, the modified DNA duplexes formed vesicular, rugby-shaped, or spherical nanostructures. Spermine and similar polyamines facilitate the assembly of DNA by mitigating the coulombic repulsion between the negatively charged DNA backbones.^38–40^

In this work, DNA conjugates with sticky phenanthrene ends were additionally modified with phenanthrene units at internal positions. After the self-assembly of the phenanthrene–DNA conjugate, the light-harvesting capabilities of the precisely arranged internal phenanthrene modification were tested. To specifically excite the internal phenanthrenes, chromophores with different absorption properties at the terminal (2,7-dialkynyl phenanthrene) and internal (3,6-dialkynyl phenanthrene) positions were used. We used 1,8-dialkynyl pyrene as an acceptor in this study, as it is reported to be an excellent acceptor for 3,6-dialkynyl phenanthrene in light-harvesting experiments inside of DNA.^37^

**Fig. 1:**
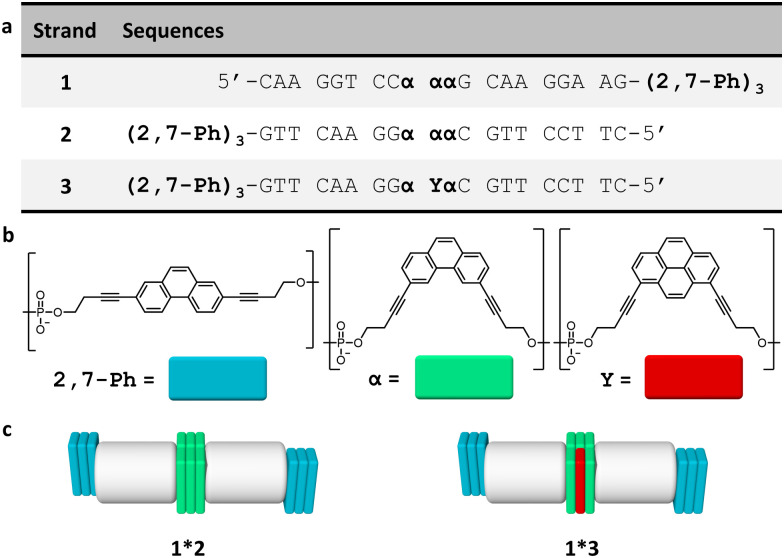
(a) Sequences of oligomers 1–3, (b) chemical structures of 2,7-dialkynyl phenanthrene (2,7-Ph), 3,6-dialkynyl phenanthrene (α), and 1,8-dialkynyl pyrene modifications (Y), and (c) illustration of the DNA duplexes formed by the hybrids 1*2 and 1*3 with DNA (grey), 2,7-Ph (blue), α (green) and Y (red).

The three DNA–chromophore conjugates 1–3 used for the study are displayed in Fig. 1. All conjugates bear internal chromophore modifications and 3′-end phenanthrene overhangs. The complementary strands 1 and 2 were both modified with three 2,7-dialkynyl phenanthrenes at the 3′-ends and three 3,6-dialkynyl phenanthrenes internally (Fig. 1b). Oligomer 3 is identical to 2 except that the midmost 3,6-dialkynyl phenanthrene is replaced by a 1,8-dialkynyl pyrene unit (Fig. 1b). The usage of two different phenanthrene isomers was chosen to allow the selective excitation of the 3,6-dialkynyl phenanthrenes in light-harvesting experiments. Preparation of oligomers 1–3 was accomplished *via* solid-phase synthesis using phosphoramidite chemistry followed by reverse-phase HPLC purification according to published procedures.^27^

**Fig. 2:**
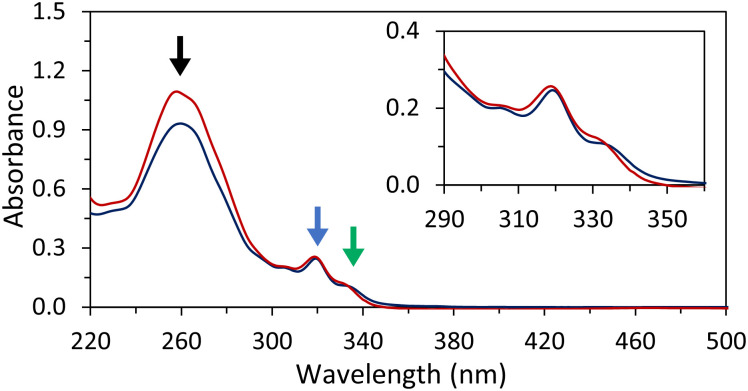
Temperature-dependent UV-vis absorption spectra of disassembled 1*2 at 75 °C (red) and self-assembled 1*2 at 20 °C (blue). Highlighted by arrows: absorption of DNA nucleobases, 3,6- and 2,7-dialkynyl phenanthrene (black), 3,6- and 2,7-dialkynyl phenanthrene absorption (blue), and 3,6-dialkynyl phenanthrene (green). Conditions: 1 μM 1*2, 10 mM sodium phosphate buffer pH 7.2, 0.10 mM spermine tetrahydrochloride, 20 vol% ethanol.

Temperature-dependent UV-vis spectra of hybrids 1*2 were recorded (Fig. 2). Hybrid 1*2 exhibits distinct 2,7-dialkynyl and 3,6-dialkynyl phenanthrene absorptions between 300 and 340 nm. Between 220 nm and 300 nm, the phenanthrene absorptions overlap with the ones of the DNA nucleobases. The peaks with maxima at 305 nm and 318 nm are attributed to both 2,7-dialkynyl and 3,6-dialkynyl phenanthrene, whereas the peak with a maximum around 330 nm originates only from the 3,6-dialkynyl phenanthrene. After cooling the hybrid 1*2 from 75 °C to 20 °C (0.5 °C min^−1^), the phenanthrene absorption bands exhibit a slight bathochromic shift (1–2 nm), whereas hypochromicity is observed for the band between 220 nm and 300 nm. In addition, a small degree of scattering was observed (inset of Fig. 2). These results indicate that the phenanthrene–DNA conjugates are aggregated at 20 °C. To further characterize the aggregation process, we measured the absorbance at 250 nm during heating and cooling (Fig. S9a, ESI‡). A nucleation temperature of 52 °C for 1*2 was determined, and hysteresis between the cooling and heating was observed. Temperature-dependent UV-vis measurements conducted with the pyrene-modified 1*3 yielded comparable results (Fig. S9b and c, ESI‡). In summary, the temperature-dependent UV-vis experiments clearly indicate aggregation of the phenanthrene–DNA conjugates upon cooling.

**Fig. 3:**
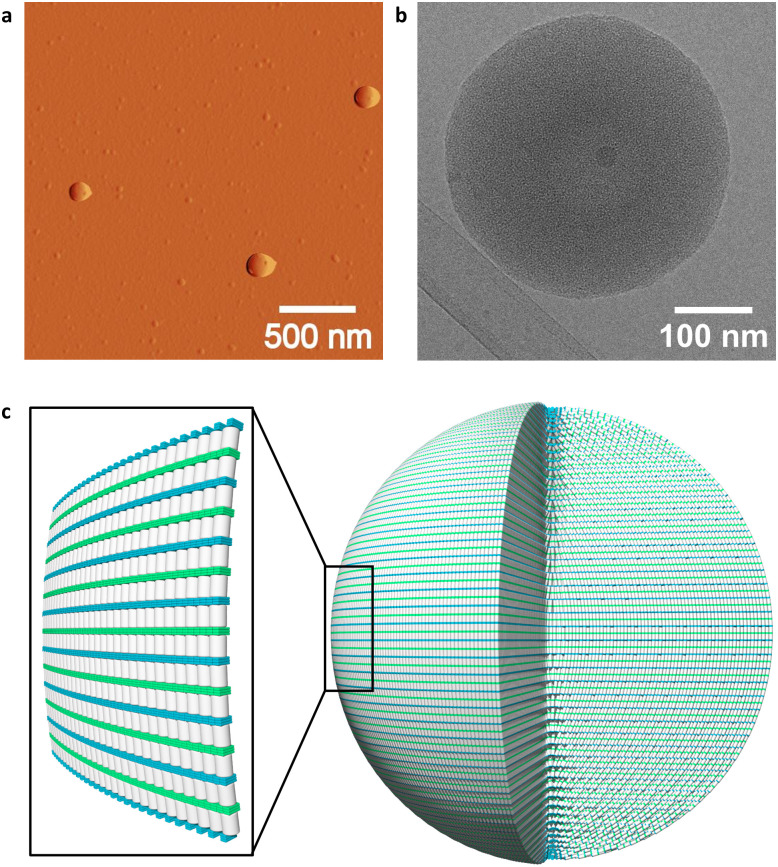
(a) AFM deflection image of 1*2 deposited on APTES-modified mica, (b) cryo-EM image of 1*2, and (c) illustration of a self-assembled 1*2 nanosphere and zoom-in on outermost layer (DNA in grey, 2,7-dialkynyl phenanthrene 2,7-Ph in blue, and 3,6-dialkynyl phenanthrene α in green). Conditions: 1 μM 1*2, 10 mM sodium phosphate buffer pH 7.2, 0.10 mM spermine tetrahydrochloride, and 20 vol% ethanol.

More detailed evidence of DNA nanostructure formation was obtained from atomic force microscopy (AFM), cryo-electron microscopy (cryo-EM), and dynamic light scattering (DLS). AFM of 1*2 on (3-aminopropyl)triethoxysilane (APTES) modified mica revealed the formation of spherical nanostructures with diameters between 150 nm and 300 nm (Fig. 3 and Fig S10, ESI‡). Spherical nanostructures with diameters of 150 nm to 350 nm were observed on cryo-EM images (Fig. 3b and Fig. S13, ESI‡). The findings of AFM and cryo-EM are furthermore in agreement with DLS measurements, which indicate a mean diameter of the spherical nanostructures of 230 ± 89 nm (Table S2, S3 and Fig. S16, ESI‡). A possible arrangement of the phenanthrene–DNA conjugates is illustrated in Fig. 3c. The nanostructures are stabilized by hydrophobic interactions between the phenanthrene sticky ends at the 3′-ends of the DNA conjugates and internal phenanthrene modifications. The coulombic repulsion of the DNA backbone is reduced by spermine tetrahydrochloride, thus stabilizing the self-assembly of the polyanionic DNA strands.

**Fig. 4:**
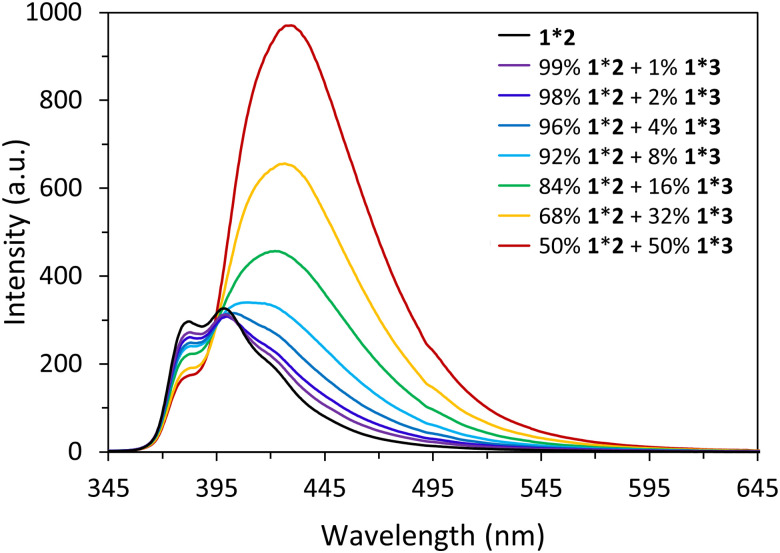
Fluorescence measurements of self-assembled nanostructures at 20 °C: 1*2 (black) and 1*3 doped spheres with 99−50% 1*2 and 1−50% 1*3 (coloured). Fluorescence quantum yields can be found in Fig. S19 ESI.‡ Conditions: 1 μM 1, 1–0.5 μM 2, 0–0.5 μM 3, 10 mM sodium phosphate buffer pH 7.2, 0.10 mM spermine tetrahydrochloride, 20 vol% ethanol, λ_ex._ 330 nm.

**Fig. 5:**
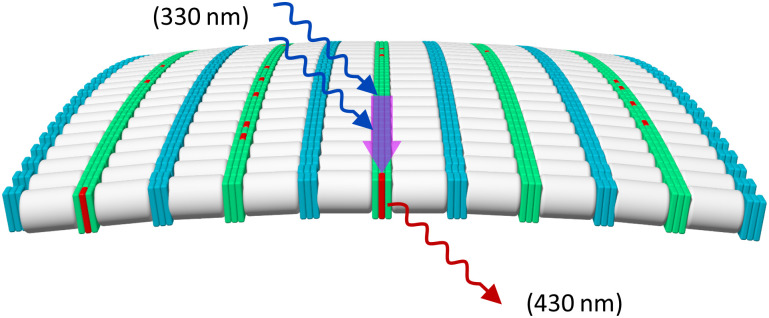
Illustration of light-harvesting process in the supramolecular assembly: excitation (blue curly arrows) of 3,6-dialkynyl phenanthrene α (light green) at 330 nm leads to FRET (purple arrow) to the 1,8-dialkynyl pyrene Y (red) resulting in phenanthrene–pyrene exciplex emission at 430 nm (red arrow). Note: the illustration is limited to a single layer of the nanosphere for simplicity; energy transfer between different layers likely occurs as well.

Fluorescence experiments were conducted to determine the light-harvesting properties of the DNA-built supramolecular nanostructures (Fig. 4 and Fig. S17, ESI‡). Selective excitation of the 3,6-dialkynyl phenanthrenes, located at internal position of 1*2, leads to phenanthrene emission at 380 nm, 400 nm, and 420 nm (Fig. 4). On the other hand, excitation of the 3,6-dialkynyl phenanthrenes in 1*3-doped (1–50%) nanostructures leads to a gradual reduction of the phenanthrene emission and the concomitant appearance and growth of a broad phenanthrene–pyrene exciplex emission with a maximum around 430 nm. The reduction of phenanthrene emission (380 nm) indicates energy transfer from 3,6-dialkynyl phenanthrene (donor) to pyrene (acceptor) in the DNA assemblies. Model calculations (ESI‡) support that the energy transfer takes place *via* a FRET (Förster resonance energy transfer) mechanism. The FRET efficiency of the doped nanospheres increase from 8% to 37% upon stepwise addition of 1*3 (1% → 50%). A value of 8% translates to approximately 50 individual 3,6-dialkynyl phenanthrene donors (equivalent to 9 individual DNA duplexes) contributing to in the harvesting process (Table S4, ESI‡). With increasing acceptor concentration, this number is continuously reduced to approximately four α units at 50% 1*3, which is expected due to the higher acceptor density. These results highlight that the internal modifications on the phenanthrene–DNA conjugate in the DNA nanospheres, the 3,6-dialkynyl phenanthrenes, can act as light-harvesting antennas by collecting and transferring excitation energy to pyrenes, resulting in phenanthrene–pyrene exciplex emission (Fig. 5).

## Conclusions

In conclusion, the supramolecular self-assembly of internally and 3′-end modified phenanthrene–DNA conjugate was described. AFM, DLS, and cryo-EM revealed the assembly of the phenanthrene–DNA conjugates into nanospheres with diameters ranging from 150 to 350 nm. The self-assembled nanospheres show light-harvesting properties. When the assemblies are doped with oligomers containing a pyrene acceptor, 3,6-dialkynyl phenanthrene units act as light-harvesting antennas. After irradiation, the excitation energy is transferred *via* FRET to the pyrene containing strand, resulting in phenanthrene–pyrene exciplex emission. FRET efficiency calculations indicate that energy is collected from up to 50 phenanthrenes and transported to a single phenanthrene–pyrene exciplex, showcasing the light-harvesting properties of the nanospheres.

## Author contributions

J. T. designed the project, performed the experiments, analysed the data, and wrote the paper. T. S. synthesized the oligomers, performed the experiments, and analysed the data. I. I. performed cryo-EM experiments, analysed the data, and contributed to the writing of the paper. S. M. L. designed the project, analysed the data, created the artwork, and contributed to the writing of the paper. B. Z. supervised the project and contributed to the writing of the paper. R. H. designed and supervised the project, analysed the data, and contributed to the writing of the paper.

## Data availability

The data supporting this article have been included as part of the ESI.‡

## Conflicts of interest

There are no conflicts to declare.

## Supplementary Material

NJ-048-D4NJ02411G-s001
