# Supplementary material for: Supramolecular assembly of phenanthrene–DNA conjugates into light-harvesting nanospheres
Source: New J Chem. 2024 Aug 30;48(36):15731–4. doi: 10.1039/d4nj02411g (PMC11367589; doi:10.1039/d4nj02411g)
Supplement: NJ-048-D4NJ02411G-s001 [file NJ-048-D4NJ02411G-s001.pdf]

Supplementary Information (SI) for New Journal of Chemistry.
This journal is © The Royal Society of Chemistry and the Centre National de la Recherche Scientifique 2024

Electronic Supplementary Information

Supramolecular Assembly of Phenanthrene-DNA Conjugates into Light-Harvesting Nanospheres

Jan Thiede,[a] Thomas Schneeberger,[a] Ioan Iacovache,[b] Simon M. Langenegger,[a] Benoît Zuber,[b] and Robert Häner*[a]

[a] Department of Chemistry, Biochemistry, and Pharmaceutical Sciences, University of Bern, Freiestrasse 3, CH-3012 Bern, Switzerland

[b] Institute of Anatomy, University of Bern, Baltzerstrasse 2, CH – 3012 Bern, Switzerland

Table of Contents

1. General Methods ... 3

2. DNA-Conjugate Synthesis and Purification ... 4

3. UV-Vis Spectroscopy ... 8

4. AFM ... 9

5. Cryo-EM ... 12

6. DLS ... 14

7. Fluorescence Spectroscopy ... 15

8. Calculations of FRET Efficiencies ... 16

9. Fluorescence Quantum Yields ... 18

10. References ... 18

1. General Methods

All reagents and solvents were purchased from commercial sources and used without further purification. The phenanthrene and pyrene phosphoramidites used in the solid-phase synthesis were synthesized according to published procedures.[1,2] Water was used from a Milli-Q system. Mass spectra were measured by the Analytical Research and Services (ARS) of the University of Bern, Switzerland, on a Thermo Fisher LTQ Orbitrap XL using Nano Electrospray Ionization (NSI). All mass spectra were measured in negative ion mode in mixtures of acetonitrile/water/triethylamine. UV-vis spectra were measured on a Jasco V-730 spectrophotometer using quartz cuvettes with an optical path of 1 cm. Fluorescence spectra were recorded on a Jasco spectrophotometer FP-8300 using an excitation and emission slit of 2.5 nm. The fluorescence quantum yield was determined according to the published procedure[3] relative to quinine sulfate in 0.5 M H_2SO_4.[4] Supramolecular self-assembly was performed *via* thermal disassembly (heating to 75 °C) and assembly (cooling 0.5 °C/min to 20 °C) in a thermostat equipped with a Peltier. Doping experiments were conducted by replacing **1*2** with **1*3** while keeping a constant hybrid concentration of 1 µM. After each addition, the nanostructures were reassembled as described above (heating to 75 °C and cooling to 20 °C) to integrate the added strands into the aggregates. Dynamic light scattering (DLS) experiments were performed on a Malvern Zetasizer Nano Series instrument (λ = 633 nm) in particle size distribution (PSD) mode (number value) at 20 °C. Atomic force microscopy (AFM) experiments were measured on a Nanosurf FlexAFM instrument under ambient conditions using tapping mode. AFM samples were prepared on (3-aminopropyl)triethoxysilane (APTES)-modified mica sheets (Glimmer “V1”, 20 mm x 20 mm, G250-7, Plano GmbH) according to published procedures.[1,5] Samples for cryo-EM were plunge-frozen using the FEI Vitrobot Mark 4 at room temperature and 100% humidity. In brief, copper lacey carbon grids were glow discharged (air -10 mA for 20 seconds). Then, 3 µL of the sample were pipetted on the girds, blotted for 3 seconds, and plunged into liquid ethane. The sample grids were stored in liquid nitrogen. Images were measured with a Gatan 626 cryo holder on a Falcon III equipped FEI Tecnai F20 in nanoprobe mode. Due to the nature of the sample, acquisition settings had to be adjusted for a low total electron dose (less than20 $e^-/Å^2$) using EPU software. The diameters of the nanostructures were measured in Fiji[6,7] using the multi-point tool to set marks.

2. DNA-Conjugate Synthesis and Purification

The phenanthrene-DNA conjugates **1**–**3** (Table S1) were synthesized on an Applied Biosystems 394 DNA/RNA synthesizer applying a cyanoethyl phosphoramidite coupling protocol on a 1 μM scale. A coupling time of 30 seconds was used for the phenanthrene and pyrene phosphoramidite as well as the DNA nucleobases (0.1 M in anhydrous acetonitrile). The solid-phase synthesis was started with a phenanthrene-modified long chain alkylamine controlled pore glass (LCAA-CPG) solid-support, which was prepared according to reported procedures.[1] After the solid-phase synthesis, **1**–**3** were deprotected and cleaved from the solid support with aqueous NH_4OH (28–30%) at 55 °C overnight. Then, the supernatants were collected, and the solid supports were washed three times with a 1:1 solution of ethanol and Milli-Q H_2O (3×1 mL). The crude phenanthrene-DNA conjugates were isolated by three rounds of lyophilization and redissolving in Milli-Q H_2O (4 ml). Afterward, the phenanthrene-DNA conjugates **1**–**3** were purified by reverse-phase HPLC (Shimadzu LC-20AT, ReproSil 100 C18, 5,0 μm, 250×4 mm) at 50 °C with a flow rate of 1 mL/min, and a detection wavelength λ of 260 nm (Solvent A: aqueous 2.1 mM triethylamine (TEA) / 25 mM 1,1,1,3,3,3-hexafluoropropan-2-ol (HFIP) pH 8; solvent B: acetonitrile) while applying the gradients listed in Table S1. The purified phenanthrene-DNA conjugates **1**–**3** were dissolved in 1 ml of Milli-Q H_2O. Subsequently, the absorbances of the conjugates at 260 nm were measured to determine the yields and the concentration of the stock solutions of **1**–**3**, using the Beer-Lambert law. For the DNA nucleobases and the pyrene and phenanthrene modifications the following molar absorption coefficients (at 260 nm) in $L \cdot mol^{-1} \cdot cm^{-1}$ were used: εA: 15'300, εT: 9'000, εG: 11'700, εC: 7'400, ε1,8-pyrene: 30'000, ε3,6-phenanthrene: 56'000, and ε2,7-phenanthrene: 47'000. The HPLC traces and mass spectra of **1**–**3** are illustrated in Figure S1–S7.

Table S1 Phenanthrene-DNA conjugate sequences **1–3**, HPLC gradients, calculated and found masses by NSI-MS, and yields.

Oligomer	Sequence (3' to 5')	HPLC gradient B [%] (t_R [min])	Calc. mass	found mass	Yield (%)
**1**	(**2,7-Ph**)$_3$-GAA GGA ACG **ααα** CCT GGA AC	10 (0), 25 (24)	7510.4653	7510.4630	18
**2**	(**2,7-Ph**)$_3$-GTT CCA GG**α αα**C GTT CCT TC	10 (0), 25 (24)	7385.3951	7385.3497	4
**3**	(**2,7-Ph**)$_3$-GTT CCA GG**α Yα**C GTT CCT TC	10 (0), 25 (24)	7409.3951	7409.3932	20

2,7-Ph: 2,7-dialkynyl phenanthrene, **α**: 3,6-dialkynyl phenanthrene, and **Y**: 1,8-dialkynyl pyrene

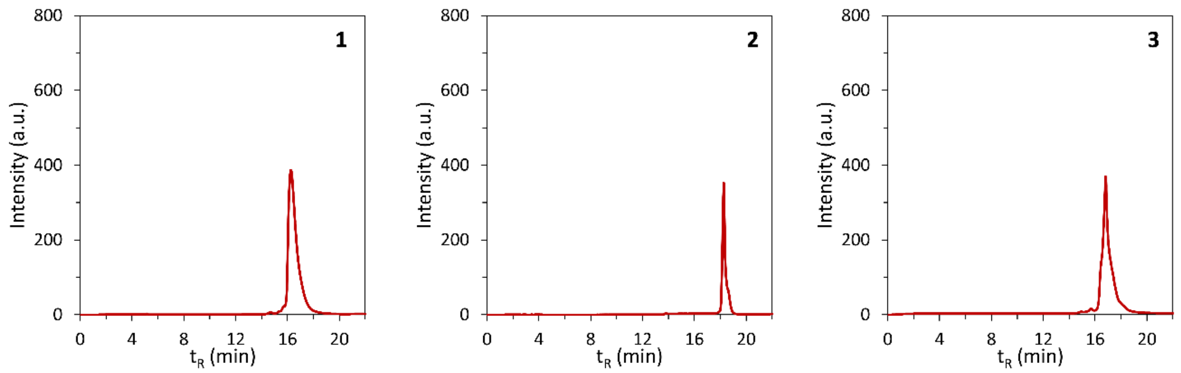

Figure S1 HPLC traces of **1**–**3** absorption measured at 260 nm.

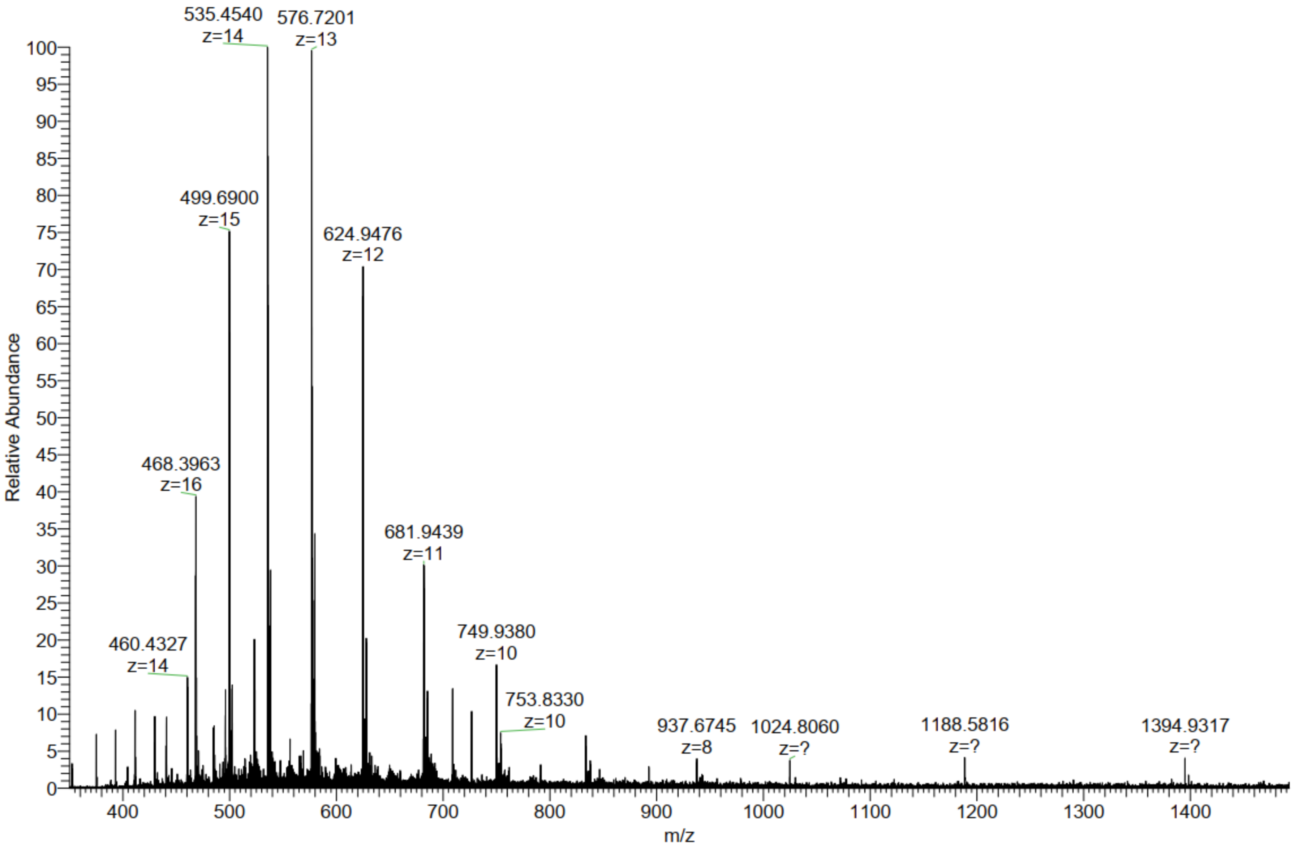

Figure S2 Mass spectrum of **1**.

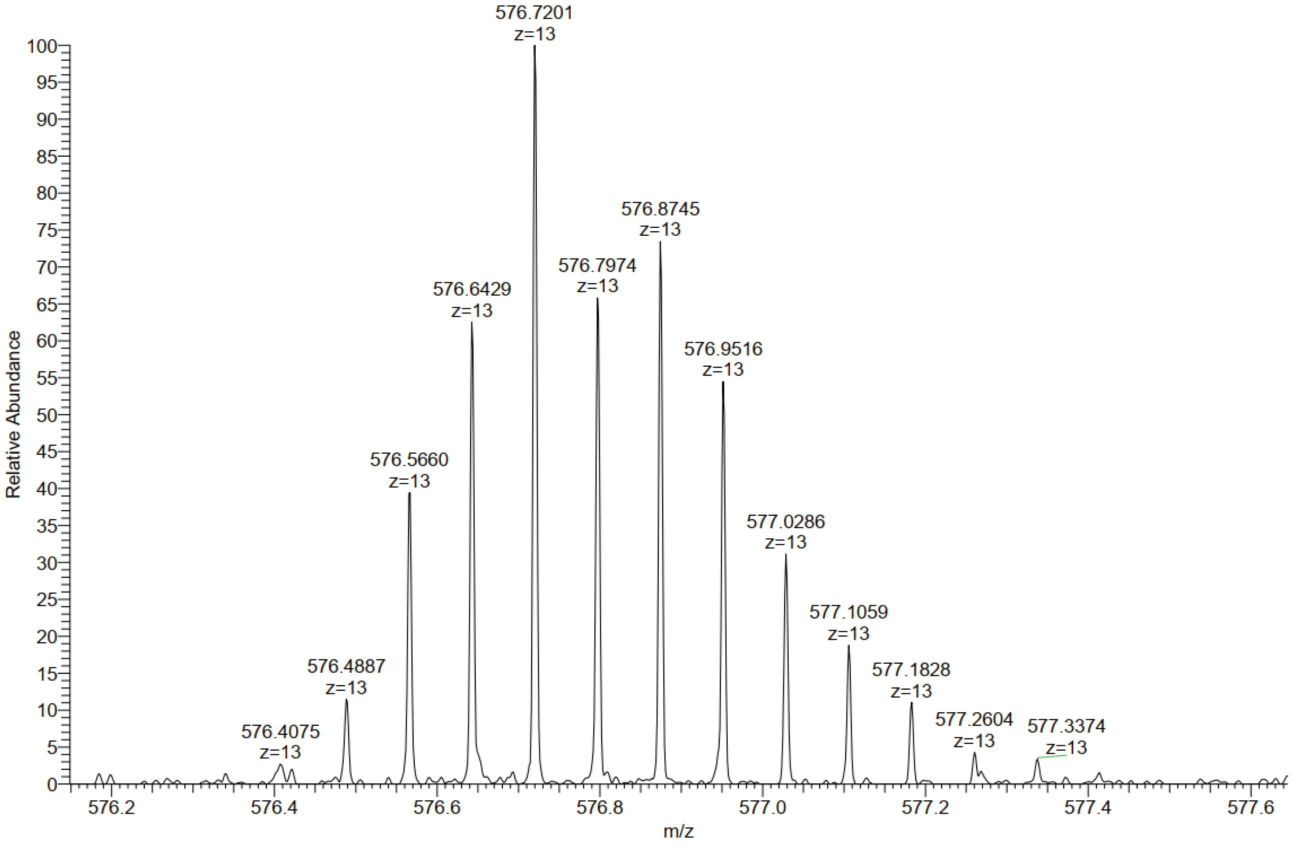

Figure S3 Mass spectrum of **1**.

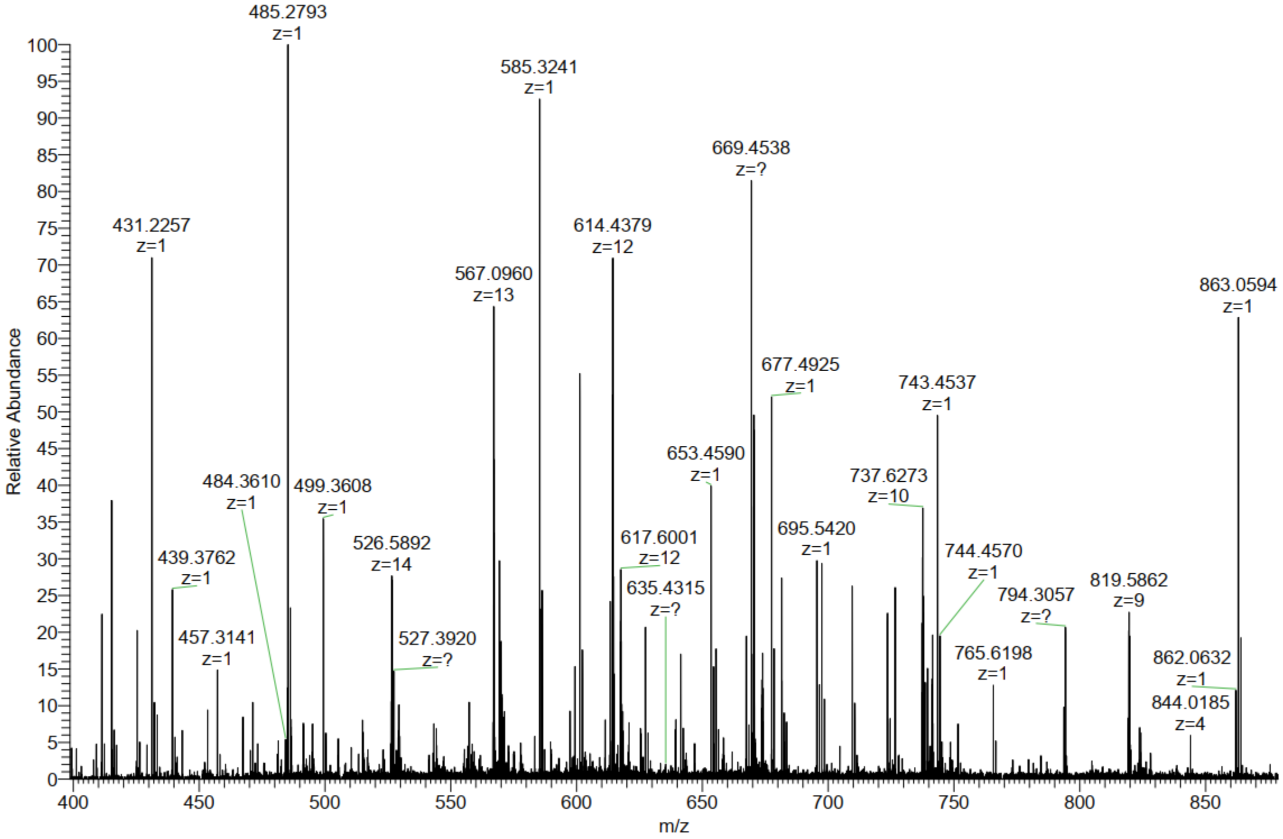

Figure S4 Mass spectrum of **2**.

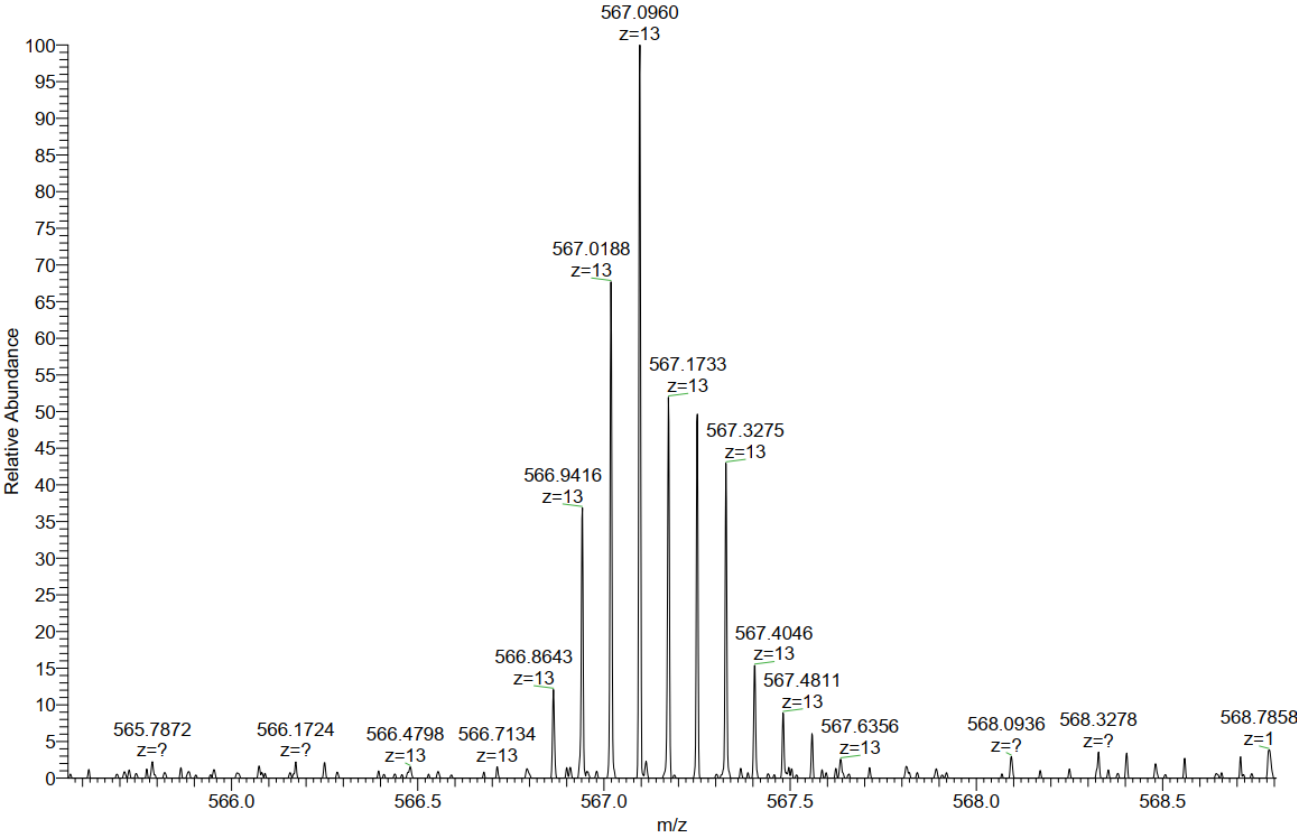

Figure S5 Mass spectrum of **2**.

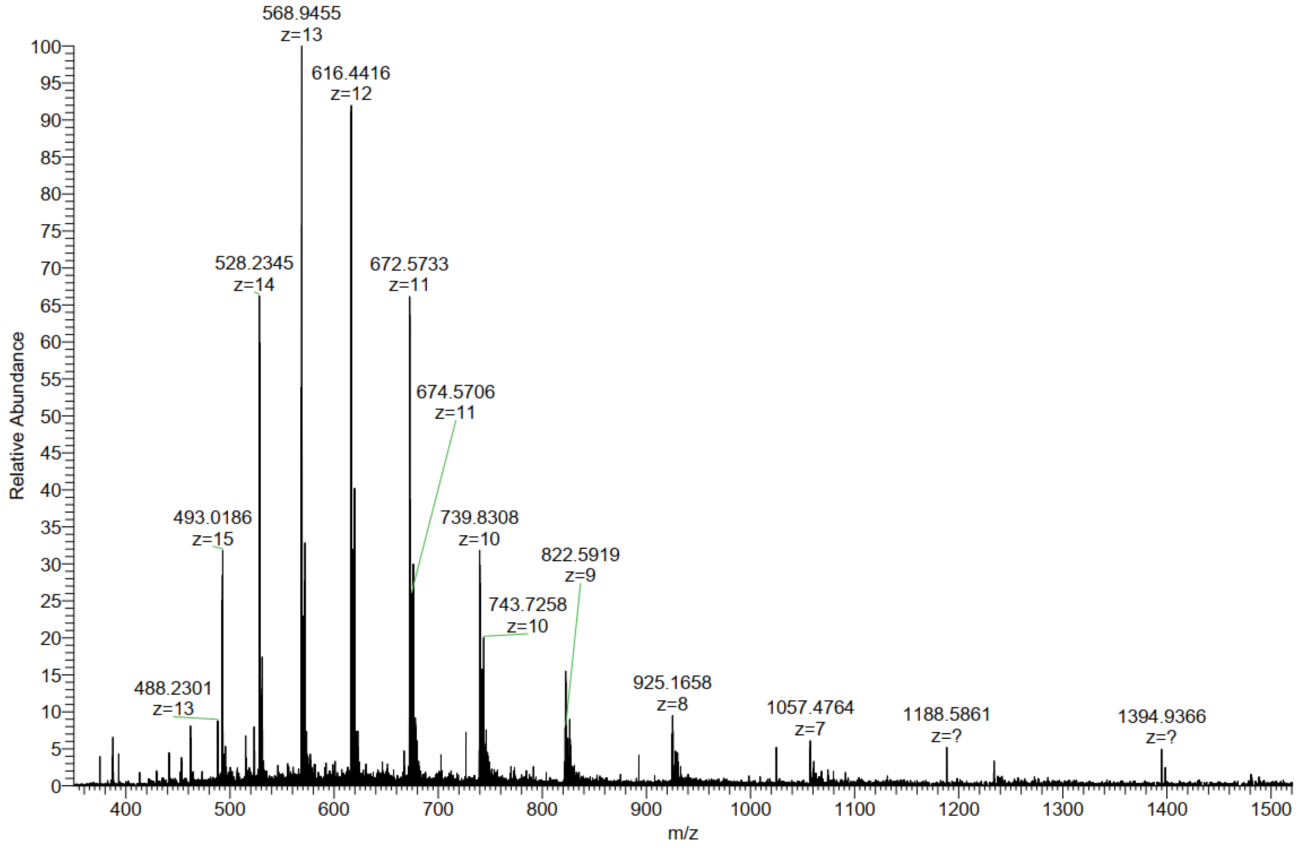

Figure S6 Mass spectrum of **3**.

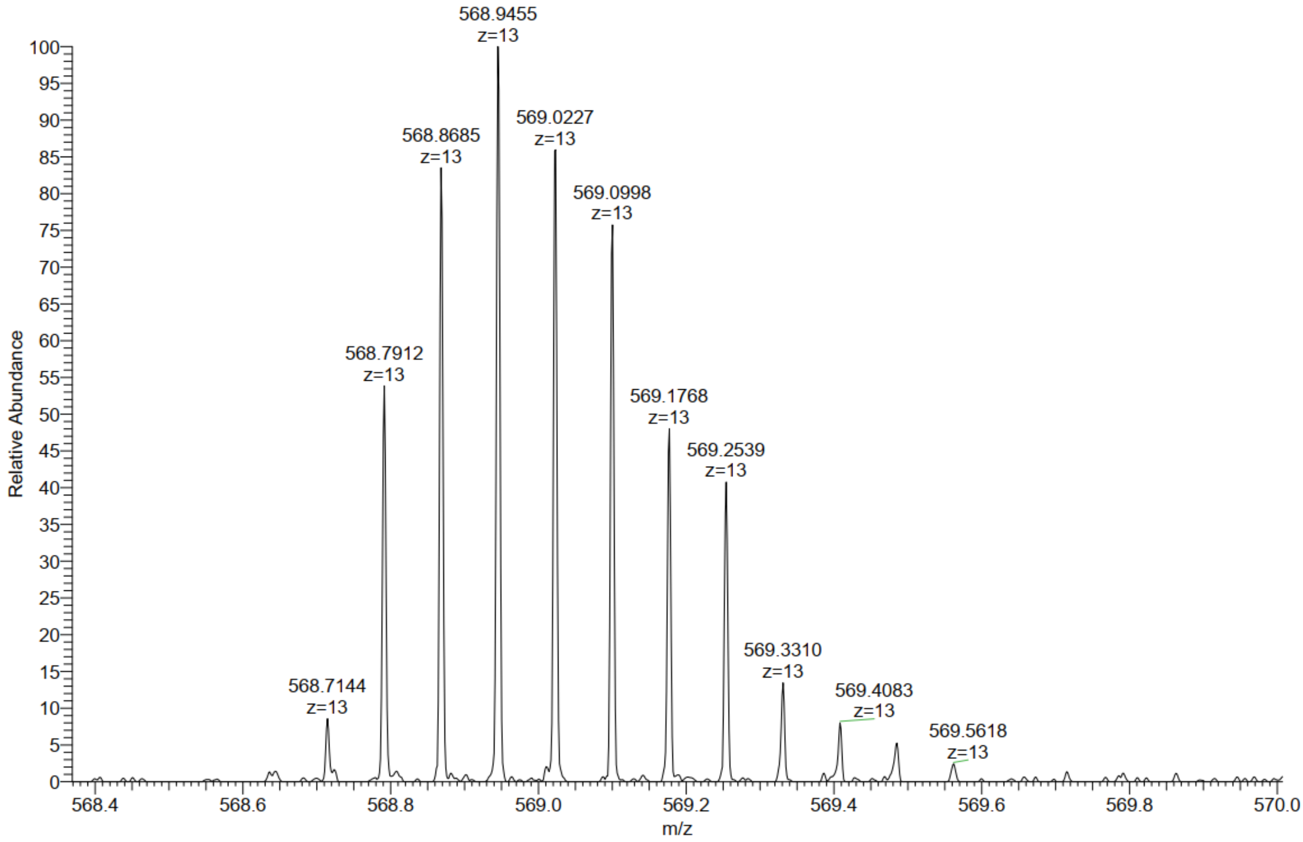

Figure S7 Mass spectrum of **3**.

3. UV-Vis Spectroscopy

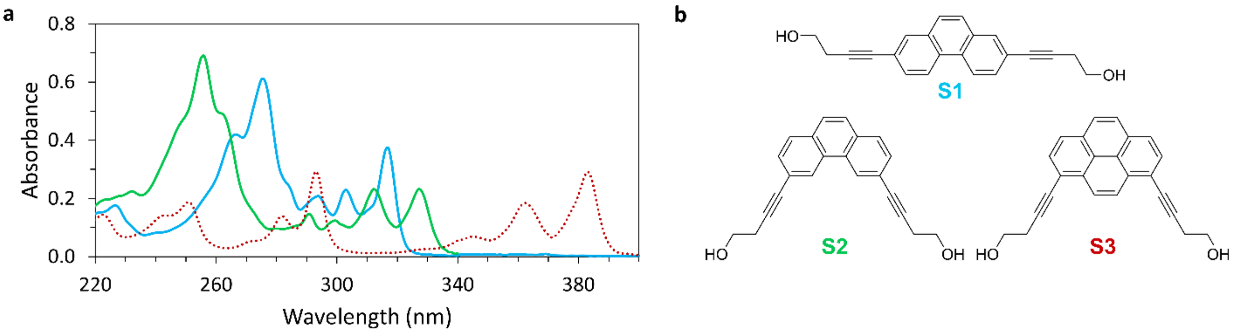

Figure S8 (**a**) UV-Vis absorption of 2,7-dialkynyl phenanthrene diol **S1**, 3,6-dialkynyl phenanthrene diol **S2**, and 1,8-dialkynyl pyrene diol **S3** and (**b**) illustration of the chemical structures of the three diols **S1–S3**. Conditions: 6 µM diols in ethanol at 20 °C.

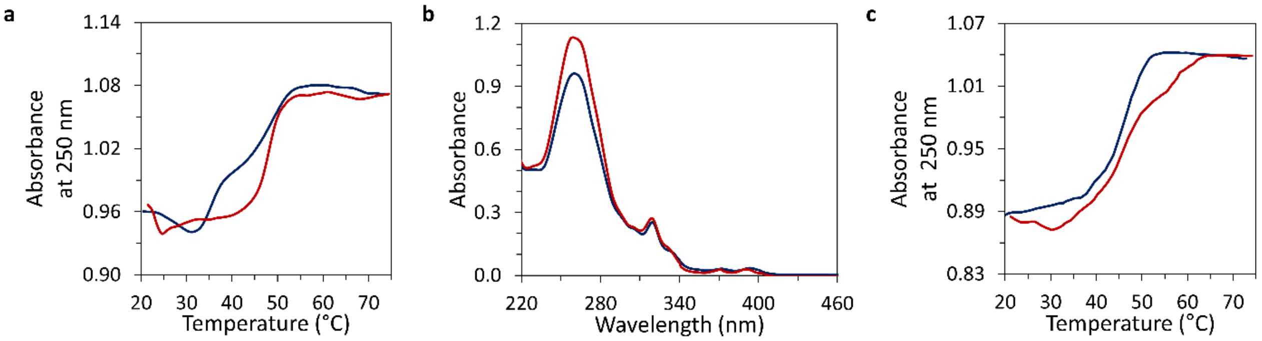

Figure S9 (**a**) UV-Vis absorption of **1*2** at 250 nm during assembly and disassembly: cooling from 75 °C to 20 °C (blue) and heating back from 20 °C to 75 °C (red) (**b**) temperature-dependent UV-vis absorption spectra of **1*3** disassembled at 75 °C (red) and self-assembly at 20 °C (blue), and (**c**) absorbance of **1*3** at 250 nm during assembly and disassembly: cooling from 75 °C to 20 °C (blue) and heating from 20 °C to 75 °C (red). Conditions: 1 µM each single strand, 10 mM sodium phosphate buffer pH 7.2, 0.10 mM spermine tetrahydrochloride, 20 vol% ethanol, heating and cooling gradient 0.5 °C·min^{-1}.

3,6-Dialkynyl phenanthrene, 2,7-dialkynyl phenanthrene and 1,8-dialkynyl pyrene units in **1*2** and **1*3** absorb at 260 nm and 250 nm (Fig. S8, UV-vis spectra of the representative diols in ethanol). Therefore, the UV-Vis absorption measurements of **1*2** and **1*3** during the assembly and disassembly process at 250 nm represent a combination of the DNA assembly and the change of absorption of the modifications due to the assembly. The measurements were conducted at 250 nm instead of the usual 260 nm for DNA hybridization to reduce the effect of the changes in the absorption by the modification.

4. AFM

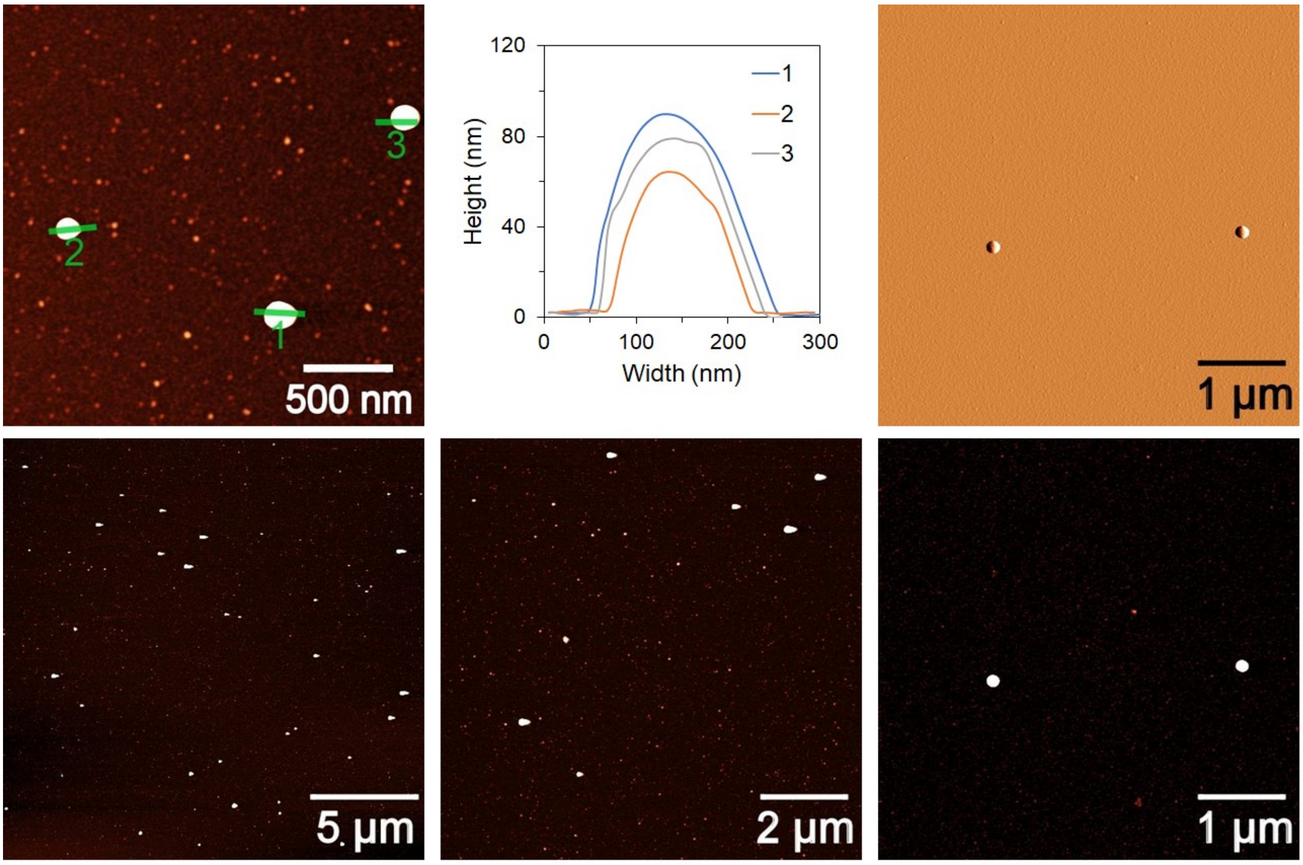

Figure S10 AFM scan (top-left) with the corresponding cross sections (top-middle), additional AFM scans (bottom), with AFM deflection scan (top-right) of **1*2** deposited on APTES-modified mica. Conditions: 1 µM each single strand, 10 mM sodium phosphate buffer pH 7.2, 0.10 mM spermine tetrahydrochloride, 20 vol% ethanol.

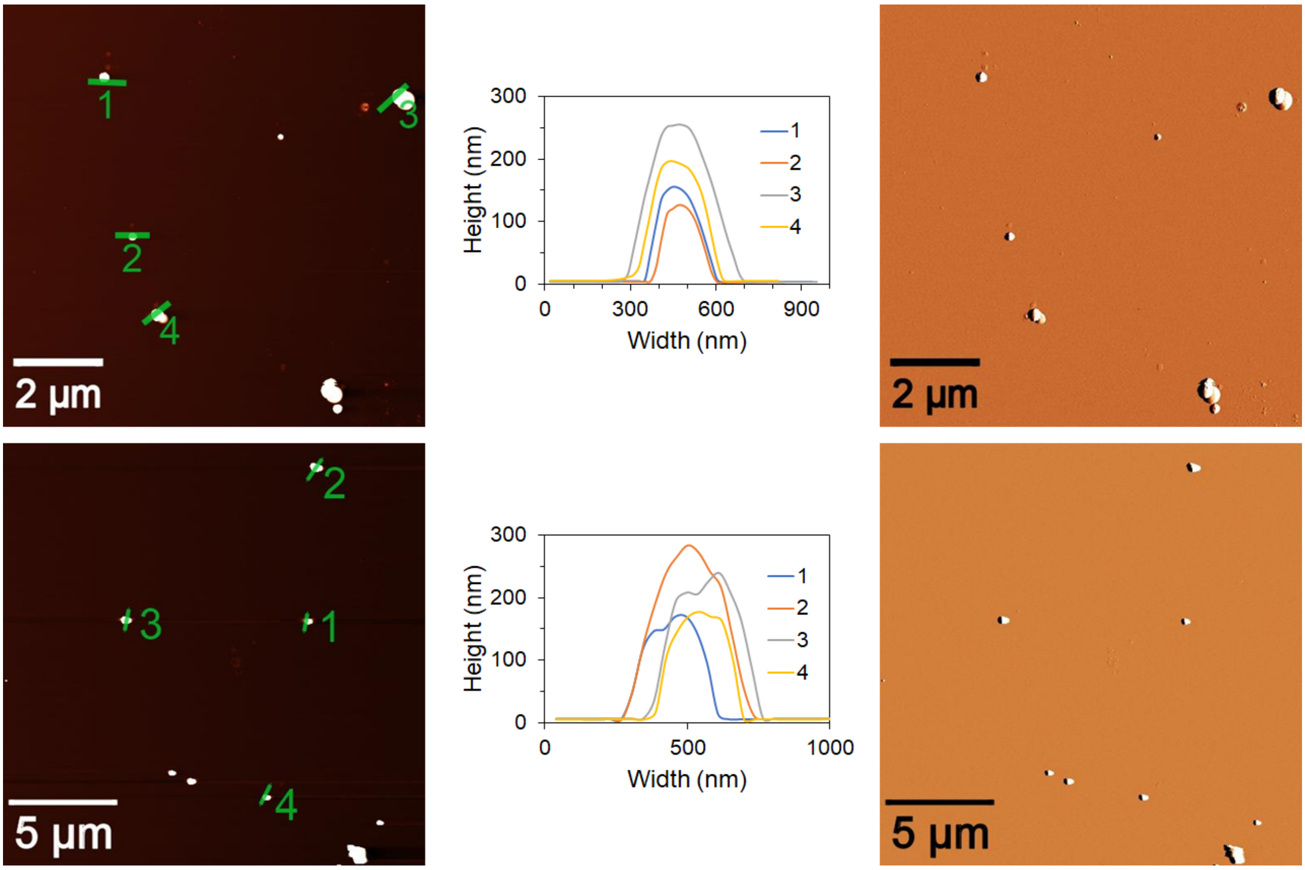

Figure S11 AFM scan (left), with the corresponding cross sections (middle), and AFM deflection scan (right) of **1*3** deposited on APTES-modified mica. Conditions: 1 µM each single strand, 10 mM sodium phosphate buffer pH 7.2, 0.10 mM spermine tetrahydrochloride, 20 vol% ethanol.

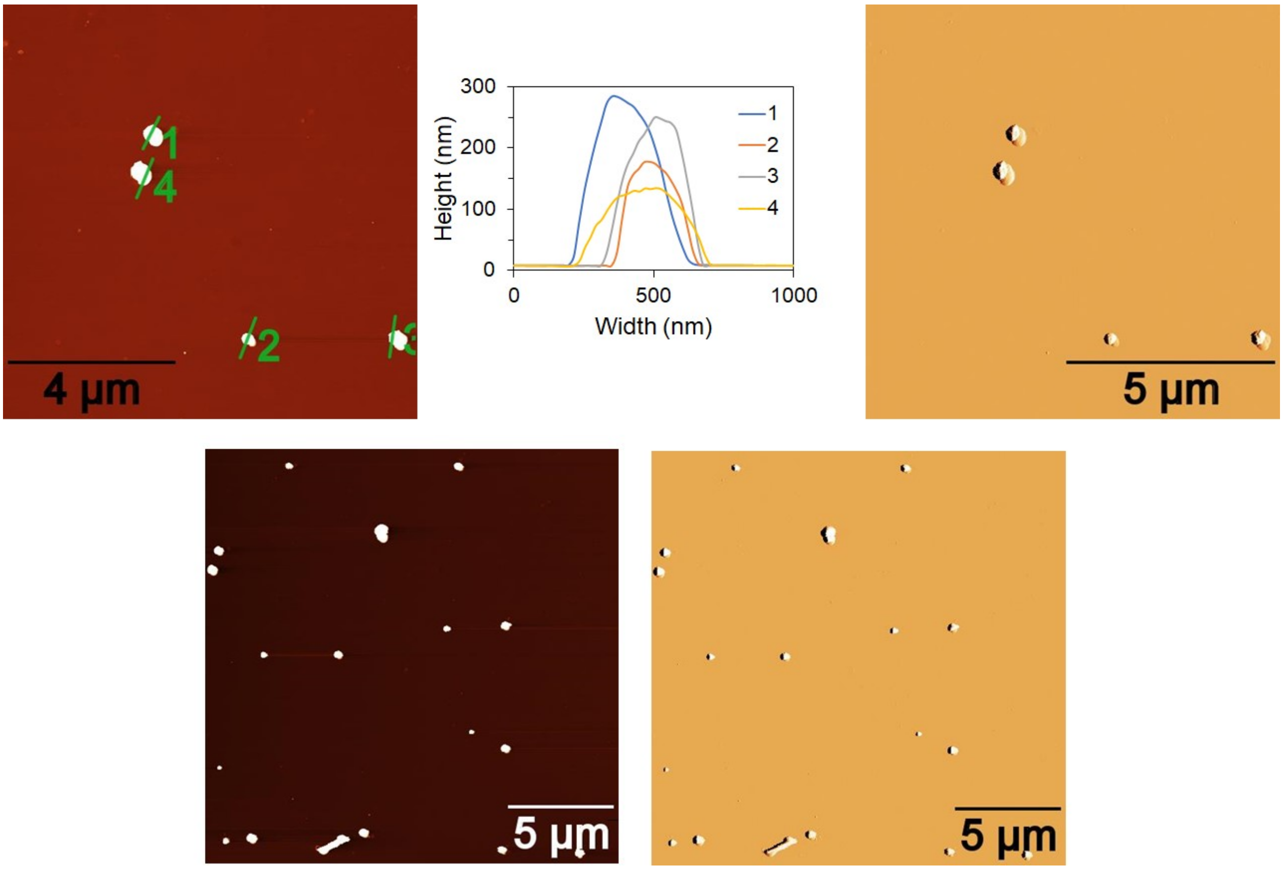

Figure S12 AFM scan (left), with the corresponding cross sections (middle), and AFM deflection scan (right) of a solution of 50% **1*2** and 50% **1*3** deposited on APTES-modified mica. Conditions: 1 μM **1**, 0.5 μM **2**, 0.5 μM **3**, 10 mM sodium phosphate buffer pH 7.2, 0.10 mM spermine tetrahydrochloride, 20 vol% ethanol.

5. Cryo-EM

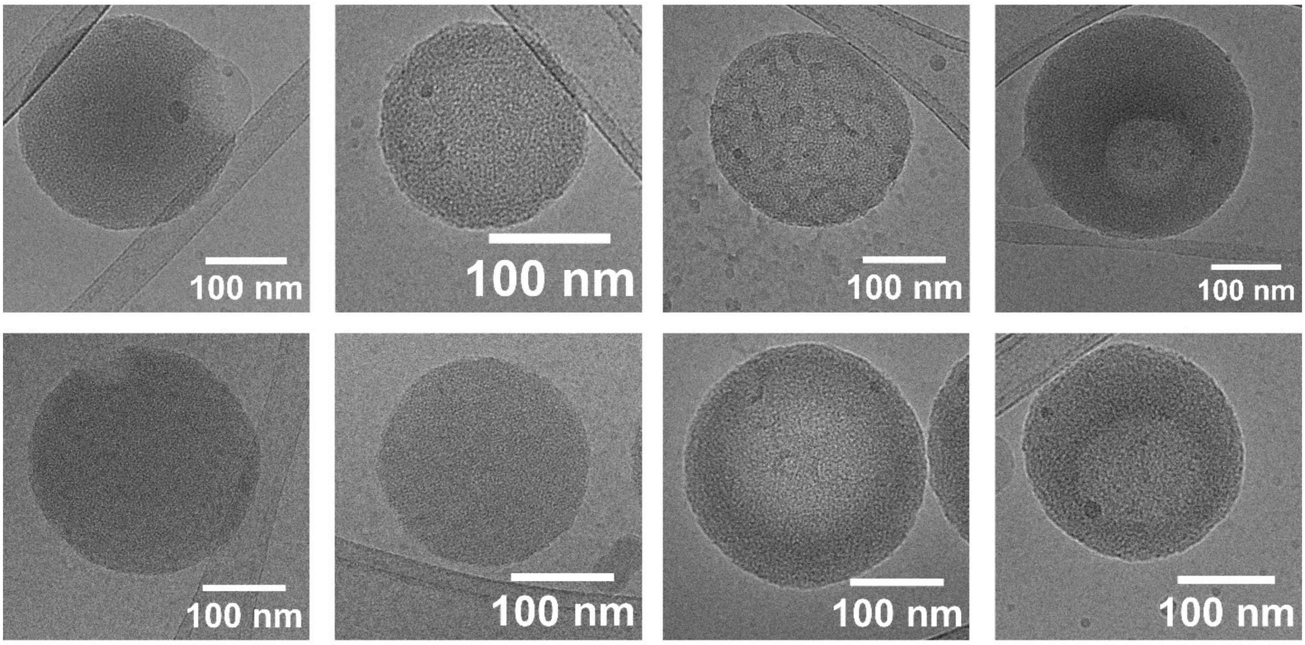

Figure S13 Cryo-EM images of **1*2**. Conditions: 1 µM each single strand, 10 mM sodium phosphate buffer pH 7.2, 0.10 mM spermine tetrahydrochloride, 20 vol% ethanol.

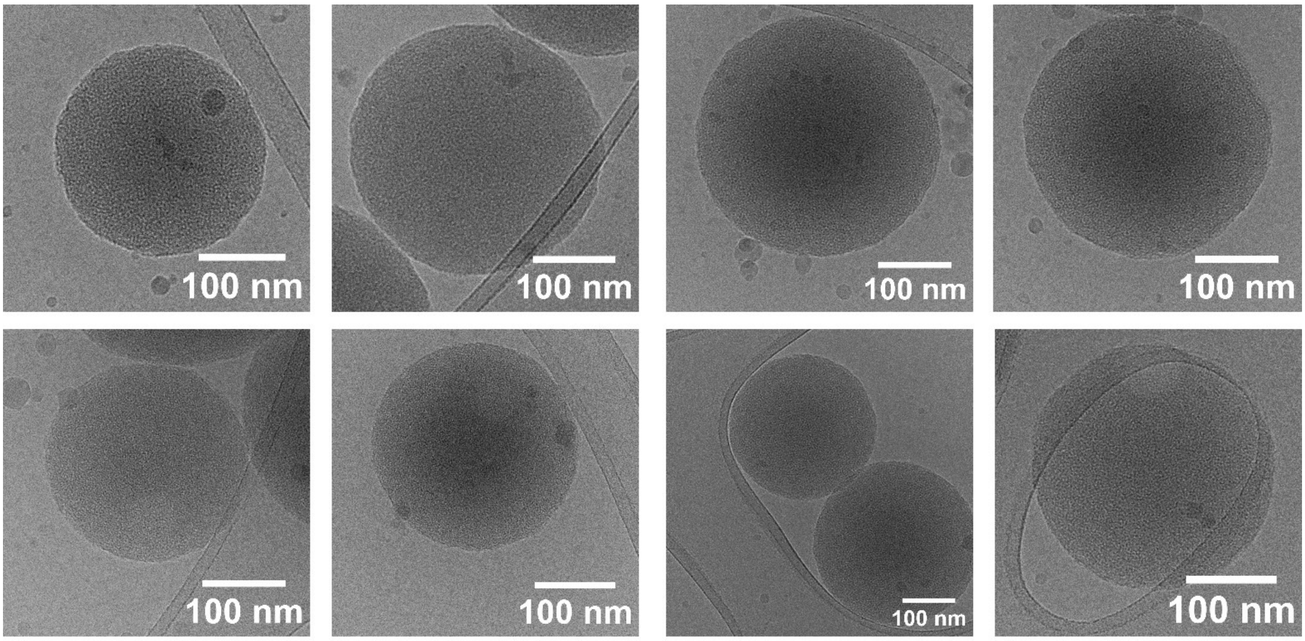

Figure S14 Cryo-EM images of **1*3**. Conditions: 1 µM each single strand, 10 mM sodium phosphate buffer pH 7.2, 0.10 mM spermine tetrahydrochloride, 20 vol% ethanol.

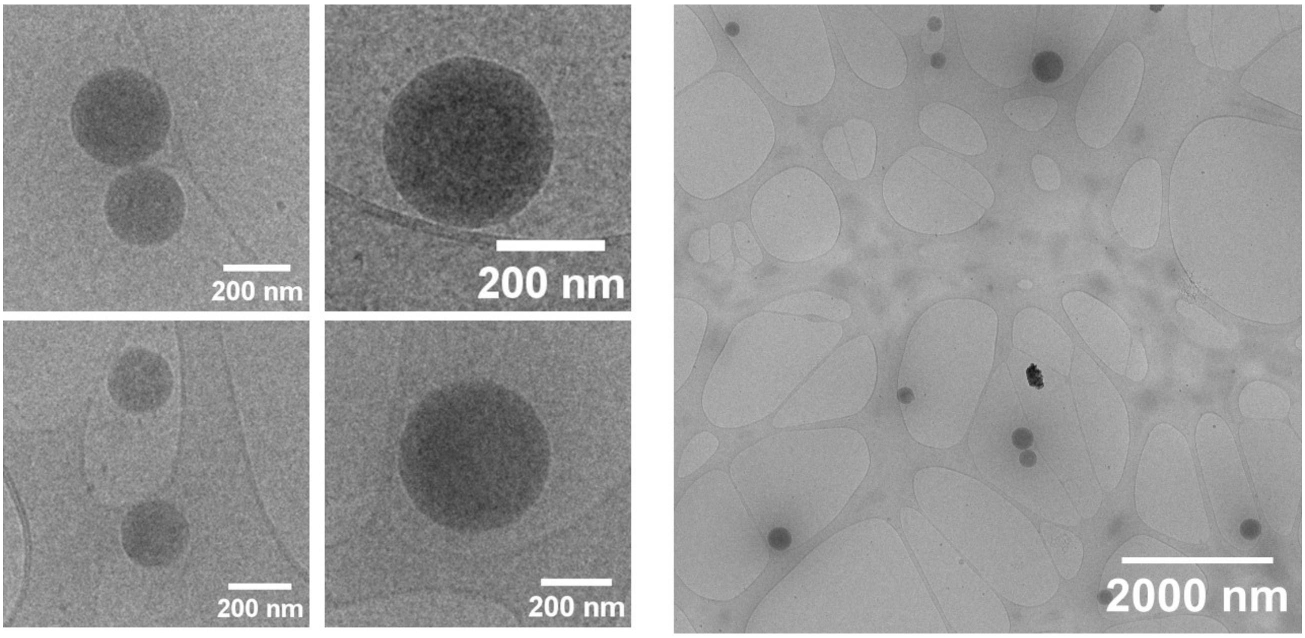

Figure S15 Cryo-EM images of 50% **1*2** and 50% **1*3**. Conditions: 1 µM **1**, 0.5 µM **2,** 0.5 µM **3**, 10 mM sodium phosphate buffer pH 7.2, 0.10 mM spermine tetrahydrochloride, 20 vol% ethanol

6. DLS

Table S2 Results and measurement conditions of DLS measurement of self-assembled **1*2**, **1*3** and 50% **1*2** with 50% **1*3** at 20 °C. Conditions: 1 µM **1** and **2**, 1 µM **1** and **3,** or 1 µM **1**, 0.5 µM **2** and 0.5 µM **3**, 10 mM sodium phosphate buffer pH 7.2, 0.10 mM spermine tetrahydrochloride, 20 vol% ethanol.

Duplex	Size Diameter (nm)	Z-Average Size Diameter (nm)	PDI	PDI width (nm)	Count Rate (kcps)	Attenuator
**1*2**	229.9 ± 88.75	208.2	0.184	89.27	17985.3	7
**1*3**	223.7 ± 97.68	183.9	0.171	75.96	18154.7	7
50% **1*2**<br>50% **1*3**	280.4 ± 142.6	217.9	0.207	99.06	15503.9	7

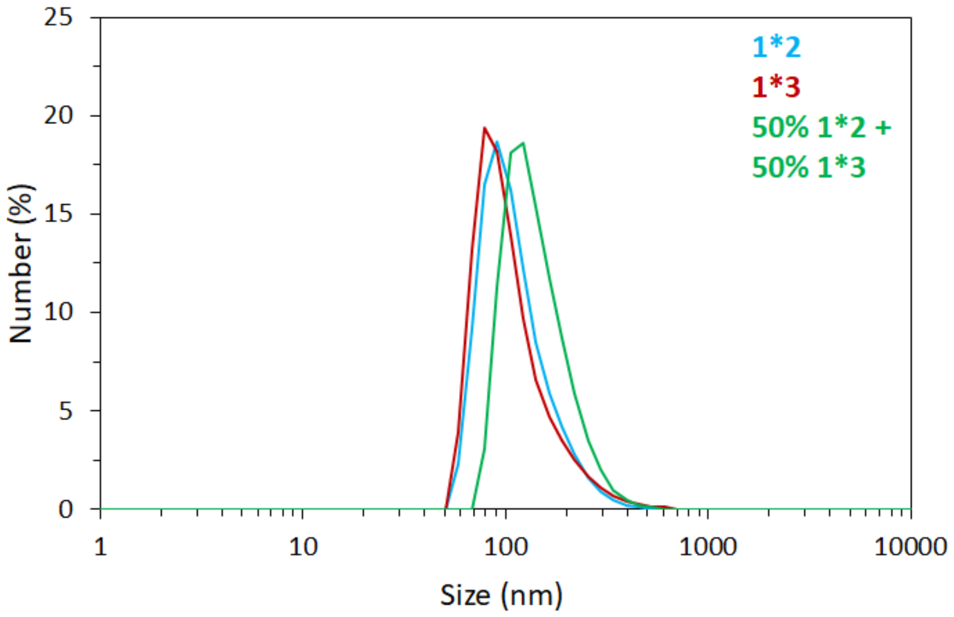

Figure S16 DLS measurements: size distribution of diameters of self-assembled **1*2** and **1*3** and 50% **1*2** with 50% **1*3** at 20 °C. Conditions: 1 µM **1** and **2**, 1 µM **1** and **3,** or 1 µM **1**, 0.5 µM **2** and 0.5 µM **3**, 10 mM sodium phosphate buffer pH 7.2, 0.10 mM spermine tetrahydrochloride, 20 vol% ethanol.

Table S3 Summary of diameters measured in cryo-EM images, AFM images and DLS. The reported distances are mean values with the corresponding standard deviation and the number of measurements (*n*) is indicated in the brackets.

Duplex	Cryo-EM Size Diameter (nm)	AFM Size Diameter (nm)	DLS Size Diameter (nm)
**1*2**	271 ± 63 (*n* = 69)	237 ± 81 (*n* = 22)	230 ± 89
**1*3**	254 ± 78 (*n* = 141)	254 ± 51 (*n* = 10)	224 ± 98
50% **1*2**<br>50% **1*3**	241 ± 82 (*n* = 123)	257 ± 103 (*n* = 10)	280 ± 142

7. Fluorescence Spectroscopy

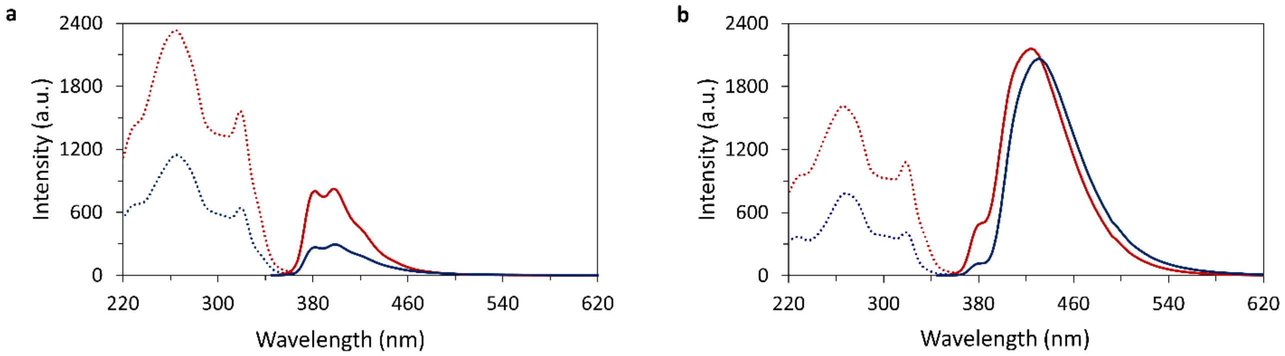

Figure S17 Fluorescence excitation (dotted) and emission (solid line) measurements of (**a**) **1*2** and (**b**) **1*3** disassembled at 75 °C (red) and assembled at 20 °C (blue). Conditions: 1 µM **1*2** or **1*3**, 10 mM sodium phosphate buffer pH 7.2, 0.10 mM spermine tetrahydrochloride, 20 vol% ethanol, $\lambda_{ex.}$ 330 nm, and $\lambda_{em.}$ 380nm.

8. Calculations of FRET Efficiencies

The light-harvesting properties of the nanostructures were determined by self-assembling different fractions of **1*2** and **1*3** (Tab. S4). The FRET efficiencies and number of donors/duplexes involved in light-harvesting to one acceptor were calculated according to the Equation S1–6 (Tab. S4). The 3,6-dialkynyl phenanthrenes (**α**) in **1*2** and **1*3** are donors, whereas the 1,8-dialkynyl pyrene (**Y**) in **1*3** are acceptors.

Equation S1 Formula used to calculate the FRET efficiencies E_{FRET}.

$$E_{FRET} = 1 - \frac{F_{DA}}{F_D}$$

- F_{DA} is the integral of the fluorescence emission of the deconvoluted donor part in presence of the acceptor
- F_D is the calculated integral of the fluorescence emission in absence of the acceptor

Equation S2 Formula used to calculate F_D.

$$F_D = \frac{F_{\mathbf{1*2}} \cdot c_D}{c_{D\mathbf{1*2}}}$$

- $F_{\mathbf{1*2}}$ is the integral of the emission of **1*2**
- c_D is the concentration of the donor
- $c_{D\mathbf{1*2}}$ is the concentration of donor in 1 µM **1*2** (6 µM)

Equation S3 Equation used to calculate c_D.

$$c_D = N_{D\mathbf{1*2}} \cdot c_{\mathbf{1*2}} + N_{D\mathbf{1*3}} \cdot c_{\mathbf{1*3}}$$

- $N_{D\mathbf{1*2}}$ is the number of donors in **1*2** (6)
- $c_{\mathbf{1*2}}$ is the concentration of **1*2**
- $N_{D\mathbf{1*3}}$ is the number of donors in **1*3** (5)
- $c_{\mathbf{1*3}}$ is the concentration of **1*3**

Equation S4 Formula to calculate the number of donors involved in FRET to one acceptor (N_D).

$$N_D = \frac{E_{FRET} \cdot c_D}{c_A}$$

- E_{FRET} is the FRET efficiency
- c_D is the concentration of donors
- c_A is the concentration of acceptors

Equation S5 Equation used to determine c_A.

$$c_A = N_{A\mathbf{1*3}} \cdot c_{\mathbf{1*3}}$$

- $N_{A\mathbf{1*3}}$ is the number of acceptors in **1*3** (1)
- $c_{\mathbf{1*3}}$ is the concentration of **1*3**

Equation S6 Formula used for the calculation of the number of duplexes involved in the excitation of one acceptor N_{Duplex}.

$$N_{Duplex} = 1 + \frac{N_D - N_{D\mathbf{1*3}}}{N_{D\mathbf{1*2}}}$$

- N_D is the number of donors involved in FRET to one acceptor
- $N_{D\mathbf{1*3}}$ is the number of donors in **1*3** (5)
- $N_{D\mathbf{1*2}}$ is the number of donors in **1*2** (6)

Table S4 At different fractions of **1*3** (c_{1*3}) and **1*2** (c_{1*2}): concentrations of donor (c_D) and acceptor (c_A), calculated fluorescence emission integrals of donor in the absence of the acceptor (F_D), deconvoluted fluorescence emission integrals of the donor in presence of the acceptor (F_{DA}), FRET efficiencies (E_{FRET}) and number of donors (**N_D**) and duplexes (**N_{Duplex}**) involved in FRET to one acceptor. All measurements are mean values of three measurements and the corresponding errors are indicated. Conditions: 0.99−0.50 µM **1*2**, 0.01−0.50 µM **1*3**, 10 mM sodium phosphate buffer pH 7.2, 0.10 mM spermine tetrahydrochloride, 20 vol% ethanol, $\lambda_{ex.}$ 330 nm.

c_{1*3} (µM)	c_{1*2} (µM)	c_D (µM)	c_A (µM)	F_D (a.u.·nm)	F_{DA} (a.u.·nm)	E_{FRET} (%)	N_D	N_{Duplex}
**0.01**	**0.99**	5.99	0.01	19465 ± 179	17858 ± 82	8.3 ± 1.8	49.5 ± 2.2	8.4
**0.02**	**0.98**	5.98	0.02	19433 ± 179	17141 ± 110	11.8 ± 2.2	35.3 ± 1.9	6.0
**0.04**	**0.96**	5.96	0.04	19368 ± 178	16313 ± 109	15.8 ± 2.2	23.5 ± 1.7	4.1
**0.08**	**0.92**	5.92	0.08	19238 ± 177	15609 ± 175	18.9 ± 3.1	14.0 ± 0.9	2.5
**0.16**	**0.84**	5.84	0.16	18978 ± 174	14441 ± 226	23.9 ± 3.9	8.7 ± 0.5	1.6
**0.32**	**0.68**	5.68	0.32	18458 ± 170	12342 ± 173	33.1 ± 3.5	5.9 ± 0.6	1.2
**0.50**	**0.50**	5.50	0.50	17873 ± 164	11349 ± 155	36.5 ± 3.3	4.0 ± 0.5	0.8

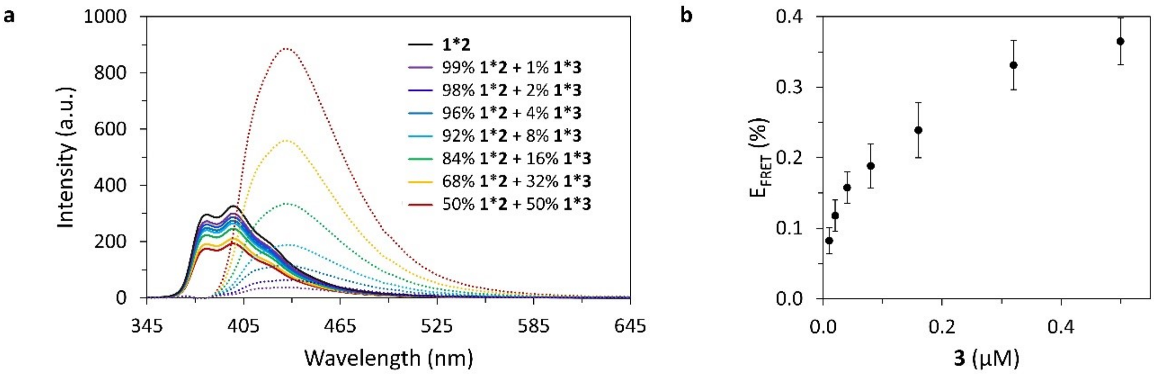

Figure S18 (a) Fluorescence emission of **1*2** (black solid line) and deconvoluted fluorescence emission parts of the donor (colored solid lines) and acceptor (colored dotted) of self-assembled **1*3** doped **1*2** at 20 °C, (b) FRET efficiencies of nanospheres containing different amounts of **1*3** (mean values of three measurements with corresponding errors). Conditions: 1 µM **1**, 1.0−0.5 µM **2**, 0.0−0.5 µM **3**, 10 mM sodium phosphate buffer pH 7.2, 0.10 mM spermine tetrahydrochloride, 20 vol% ethanol, $\lambda_{ex.}$ 330 nm.

9. Fluorescence Quantum Yields

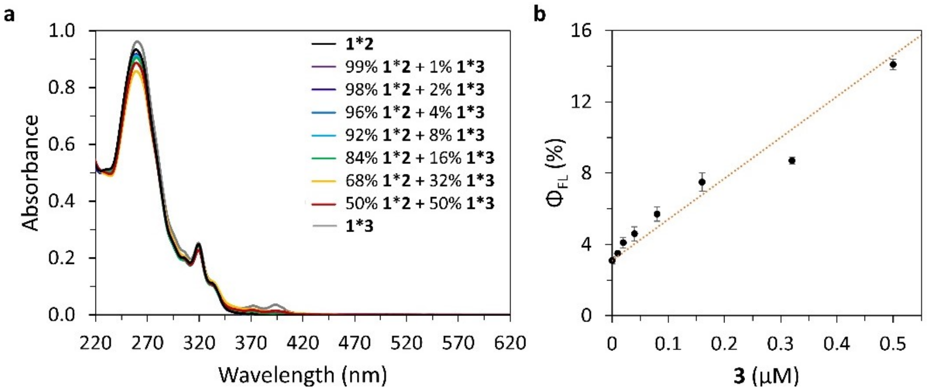

c

**1*3** (μM)	**1*2** (μM)	Φ_{FL} (%)
**0**	**1**	3.1 ± 0.2
**0.01**	**0.99**	3.5 ± 0.1
**0.02**	**0.98**	4.1 ± 0.3
**0.04**	**0.96**	4.6 ± 0.4
**0.08**	**0.92**	5.7 ± 0.4
**0.16**	**0.84**	7.5 ± 0.5
**0.32**	**0.68**	8.7 ± 0.2
**0.50**	**0.50**	14.1 ± 0.3
**1.00**	**0**	26.1 ± 0.3

Figure S19 (a) UV-vis absorption spectra of self-assembled **1*2** (black), **1*3** (grey), and 1–50% **1*3** doped **1*2** (colored) at 20 °C used for the calculation of the fluorescence quantum yields, (b) fluorescence quantum yields (**Φ_{FL}**) of self-assembled **1*2** and 1–50% **1*3** doped **1*2** (black dots) and expected calculated quantum yield (orange dashed line), and (c) table of the fluorescence quantum yields, mean values of three measurements with the corresponding errors. Conditions: 1 µM **1**, 1.0–0 µM **2**, 0–1.0 µM **3**, 10 mM sodium phosphate buffer pH 7.2, 0.10 mM spermine tetrahydrochloride, 20 vol% ethanol, $\lambda_{ex.}$ 330 nm.

As expected, upon doping **1*2** with **1*3**, the fluorescence quantum yields progressed linearly to the quantum yield of **1*3.**

10. References

1 C. D. Bösch, S. M. Langenegger and R. Häner, *Angew. Chem. Int. Ed.*, 2016, **55**, 9961–9964.

2 C. B. Winiger, S. Li, G. R. Kumar, S. M. Langenegger and R. Häner, *Angew. Chem. Int. Ed.*, 2014, **53**, 13609–13613.

3 W. H. Melhuish, *J. Phys. Chem.*, 1961, 7.

4 S. Fery-Forgues and D. Lavabre, *J. Chem. Educ.*, 1999, **76**, 1260.

5 S. Rothenbühler, I. Iacovache, S. M. Langenegger, B. Zuber and R. Häner, *Nanoscale*, 2020, **12**, 21118–21123.

6 M. Linkert, C. T. Rueden, C. Allan, J.-M. Burel, W. Moore, A. Patterson, B. Loranger, J. Moore, C. Neves, D. MacDonald, A. Tarkowska, C. Sticco, E. Hill, M. Rossner, K. W. Eliceiri and J. R. Swedlow, *J. Cell Biol.*, 2010, **189**, 777–782.

7 J. Schindelin, I. Arganda-Carreras, E. Frise, V. Kaynig, M. Longair, T. Pietzsch, S. Preibisch, C. Rueden, S. Saalfeld, B. Schmid, J.-Y. Tinevez, D. J. White, V. Hartenstein, K. Eliceiri, P. Tomancak and A. Cardona, *Nat Methods*, 2012, **9**, 676–682.
